# Deuxième maladie de Köhler

**DOI:** 10.11604/pamj.2014.18.189.4785

**Published:** 2014-07-04

**Authors:** Issam Elouakili, Younes Ouchrif, Redouane Ouakrim, Mohammed Kharmaz, Farid Ismael, Mly Omar Lamrani, Abdou Lahlou, Ahmed El Bardouni, Mustapha Mahfoud, Mohamed Saleh Berrada, Mouradh El Yaacoubi

**Affiliations:** 1 Service de Traumatologie-Orthopédie, CHU Ibn Sina, Rabat, Maroc

**Keywords:** Ostéotomies, nécrose avasculaire, métatarsalgie, Osteotomy, avascular necrosis, metatarsalgia

## Abstract

Les auteurs rapportent 17 cas de la deuxième maladie de Köhler. Il s'agit de jeunes femmes (13 cas), qui ont consulté pour des douleurs métatarso-phalangiennes unilatérales de type mécanique. La maladie touche la deuxième tête métatarsienne dans 14 cas. Le diagnostic est posé grâce à la radiologie: le stade III est retrouvé dans 11 cas. Nous avons opté pour l'intervention de Gauthier: Ostéotomie de dorsiflexion de la tête métatarsienne par résection cunéiforme à base dorsale de la zone nécrotique. Les suites opératoires sont bonnes et l’évolution est favorable pour nos patients, avec un recul moyen de 3 ans.

## Introduction

La deuxième maladie de Kohler est une ostéonécrose aseptique des têtes métatarsiennes à leur partie dorsale. Cette affection est aussi connue sous le terme d'ostéochondrite ou maladie de Freiberg. Elle touche avec prédilection la femme et débute pendant l'adolescence, bien que rare, elle est la quatrième des nécroses avasculaires les plus communes, touchant les surfaces articulaires [1]. ‘L’étiologie de cette affection est peu connue, certes un facteur vasculaire ischémique a été évoqué mais son substratum réel demeure imprécis. Plusieurs techniques chirurgicales ont étés décrites dans le traitement de la maladie de Freiberg. L'ostéotomie de dorsiflexion par résection cunéiforme de la zone nécrotique décrite et rapportée par Gauthier dans le 48^ème^ rapport de la SOFCOT semble intéressante [2, 3]. Nous nous proposons ici de la présenter et d’évaluer ses résultats à travers notre expérience sur une série de 17 cas.

## Méthodes

Nous rapportons une série de 17 patients présentant une maladie de Freiberg colligés à la clinique Universitaire de Traumatologie-Orthopédie du CHU de Rabat. On note une certaine prédominance dans la deuxième décade de la vie (12 cas); particulièrement entre 16 et 20 ans, mais il existe également des cas chez l'adulte: 2 cas entre 40 et 60 ans. La prédominance du sexe féminin est manifeste (13 cas). Le symptôme initial est toujours une métatarsalgie ponctuelle à la marche d'intensité variable. Cette douleur survient à l'appui principalement lors du passage du pas, elle disparaît au repos. Le morphotype du pied était de type égyptien dans 12 cas et carré dans 5 cas. L'examen note une douleur provoquée à la pression dorsale ou plantaire de la tête métatarsienne (1'4 cas), exacerbée lors de la mobilisation en flexion dorsale forcée ou en compression axiale de l'articulation atteinte. Dans 3 cas, un hallux valgus débutant a été noté.

**Figure 1:**
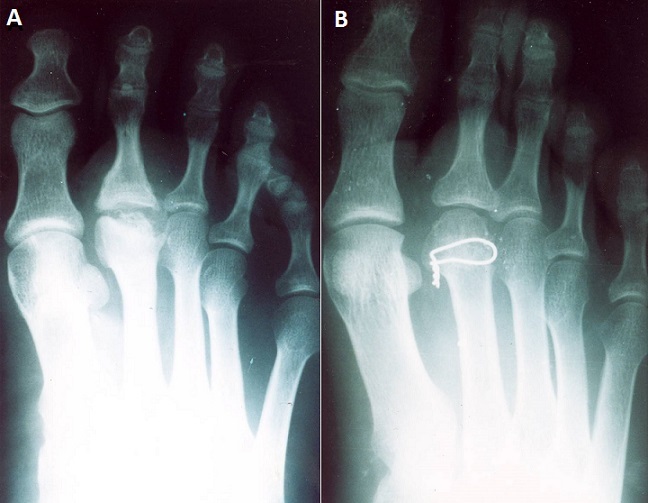
a) Ostéochondrite de la tête du 2^ème^ métatarsien stade 3 chez un patient de 20 ans. Noter l'aspect massif de la diaphyse de M2; b)Le même cas à 3 ans de recul d'une ostéotomie de Gauthier. Aspect quasi normal de la tête de M2 et de la MP

Les radiographies du pied de face, profil et les trois quarts nous ont permis d'abord de reconnaître le siège de l'ostéochondrite (Figure 1 (a), Figure 1 (b)): dans 3 cas, le 3^ème^ métatarsien était concerné. Dans tous les autres, c’était le 2^ème^. Etait analysé ensuite le morphotype métatarsien en étudiant la parabole métatarsienne à la recherche d'un vice architectural: nous n'avons pas noté en particulier d'excès de longueur de M2 par rapport à M1 (index-plus-minus M1= M2) ou aux autres rayons (longueur décroissante). Un aspect massif de M2 avec hypertrophie de la diaphyse a été noté dans 13 cas, témoin de la surcharge de ce rayon. Le 3^ème^ métatarsien était du même aspect dans le cas ou il était atteint. En plus de ces signes, nous avons distingué selon la classification radiologique de Smillie et Axhauser [4]: Stade II: 2 cas; Stade III: 11 cas; Stade IV: 4 cas. Nous n'avons pas eu de cas de stade I, trop précoce et rarement dépisté, ni de stade V ou la nécrose très importante est associée à l'arthrose. Il n'y avait pas de luxation de la MP du rayon concerné.

Le 1er métatarsien est court en varus du faite l'hallux valgus dans les 3 cas signalés, mais avec un angle M1-M2 modéré. Sous anesthésie générale ou spinale, garrot pneumatique à la racine du membre; l'intervention est menée par voie dorsale en regard de l'articulation métatarso-phalangienne en passant entre tendon extenseur et pédieux. Une arthrolyse est systématiquement pratiquée. On perfore à la broche deux trous de part et d'autre de la métaphyse. Un fil d'acier est passé à travers ces deux trous en cadre. Une ostéotomie est réalisée par résection cunéiforme à base dorsale emportant la zone nécrotique sous ostéochondrale mais respectant la charnière cartilagineuse plantaire qui est simplement pliée. Le montage est assuré par le fil d'acier qui est serré. L'appui est repris dés les premiers jours. Dans les 3 cas d'hallux valgus, une cure de celui-ci selon Mac Bride modifié (libération latérale de la MP du 1er rayon, exostosectomie et transfert du court abducteur) a été associée. Aucune ostéotomie d'accourcissement, en particulier sur M2 n'a été associée.

## Résultats

**Figure 2:**
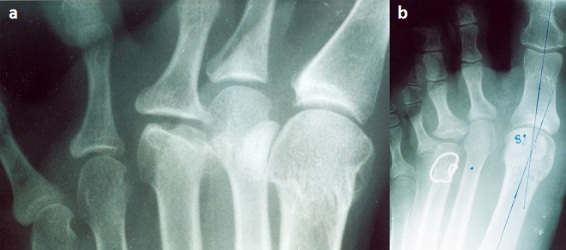
a) Ostéochondrite de la tête du 3^ème^ métatarsien stade 4 chez une patiente de 34 ans, associée à un Hallux valgus. Aspect massif de la diaphyse de M3; b)Résultat favorable à 2 ans après ostéotomie de Gauthier de M3 et cure de l'hallux valgus

Sur le plan anatomique, nous n'avons pas eu de démontage et la consolidation de l'ostéotomie a toujours été obtenue La congruence radiologique de l'articulation métatarso-phalangienne a été satisfaisante dans 12 cas. L'harmonie de la parabole métatarsienne a été respectée dans tous les cas puisque l'ostéotomie n'avait pas d'effet raccourcissant sur le plan clinique (Figure 2 (a), Figure 2 (b)), et en fonction de la disparition de la métatarsalgie, de la conservation de la mobilité de l'articulation métatarso-phalangienne et de la fonction de l'orteil en particulier lors du déroulement du pas à la marche, nous avons eu 13 bons résultats. Avec un recul moyen de 3 ans (2 à 5 ans), nous n'avons noté aucun cas de métatarsalgies au niveau du même rayon ni au niveau des rayons voisins. Nos résultats étaient donc constamment satisfaisants.

## Discussion

L'atteinte de la 2éme tête métatarsienne a été observée chez 14 malades. L'ensemble des publications font état de cette localisation préférentielle, ce métatarsien est le plus long, le plus rigide et le moins mobile en raison de son encastrement entre deux cunéiformes et deux métatarsiens. Moins fréquente est l'atteinte du 3éme métatarsien (3 cas dans notre série), exceptionnelle est celle du 4éme. La localisation au niveau du 5^ème^ métatarsien est possible, un cas a été publié en 1991 [5].

Le traitement de la maladie de Freiberg au stade précoce doit chez le jeune adolescent jouer la carte de la guérison spontanée. Une semelle plâtrée avec immobilisation stricte de la métatarso-phalangienne en légère flexion plantaire et suivi radiologique régulier de l’évolution est la thérapeutique de choix [6]. En cas d’échec ou chez les sujets plus âgés ou vu tardivement, ou lorsque la forme de la tête est déjà déformée, il est conseillé d'opérer [3, 6]. Le but du traitement chirurgical [7] est de soulager l'hyperpression sous la tête du 2éme métatarsien sans entraîner un déséquilibre statique qui amènera une métatarsalgie au niveau des rayons voisins [8]. L'ostéotomie du col du métatarsien par résection d'un coin osseux à base dorsale selon la technique décrite par Gauthier [2] n'ayant pas d'effet raccourcissant répond à cette condition.

Aux stades II, III et IV de Smilie [4], l'ostéotomie de flexion dorsale est la technique indiquée [6, 9–11]. Les résultats ont toujours été satisfaisants sur le plan fonctionnel et les suites simples et rapides. La reprise de la marche avec appui s'effectue en moyenne au bout de 2 à 4 jours sauf intervention associée. La reprise d'une activité normale est obtenue dans un délai de 5 à 6 semaines. Quant au stade V de Smillie, vu l'importance des lésions anatomiques et la fréquence de l'arthrose métatarso- phalangienne, le traitement conservateur se discute. La résection de la tête métatarsienne (arthroplastie modulante) est abandonnée [7, 8] à cause de ses inconvénients d'instabilité majeure du clavier métatarsien. Par contre, l'intervention de Gauthier reste indiquée à ce stade tardif, mais trouve sa limite quand la nécrose de la tête métatarsienne est subtotale, sans zone inférieure saine susceptible d’être ramenée par l'ostéotomie en position fonctionnelle. Dans ce cas se pose l'indication de remplacement prothétique (prothèse de Swanson). Il en est de même, en cas d'arthrose métatarso-phalangienne avec raideur articulaire très importante.

Dans 3 cas, nous avons traité un hallux valgus modéré associé, par une double stabilisation active et passive de MP1 du 1er rayon (libération externe, capsulorraphie interne et transfert du court abducteur). Les résultats étaient bons. L'association hallux valgus-maladie de Freiberg est classique [6–8]. La maladie de Freiberg est considérée comme la conséquence de l'insuffisance géométrique du 1er rayon dont les charges sont transférées sur le second. Son traitement s'impose [6–8]. Seules ses modalités se discutent: ostéotomie métatarsienne (Scarf) pour certains [7, 8], ostéotomie d'accourcissement de P1 et stabilisation activo-passive pour d'autres [6].

## Conclusion

La maladie de Freiberg est une affection à laquelle il faut penser chez tout patient qui consulte pour des douleurs mécaniques de l'avant pied. Le diagnostic est essentiellement radiologique. L'ostéotomie du col du 2^ème^ métatarsien est une méthode conservatrice, simple, utilisable dans la majorité des cas. Elle semble donner des résultats satisfaisants avec disparition des douleurs de l'avant pied et sans récidive puisqu'elle n'induit pas de vice architectural. L'hallux valgus, fréquemment associé doit être recherché et traité. Le port de talon haut reste définitivement proscrit.

## References

[1] Bineck G (1988). Freiberg disease complicating unreleated trauma. Orthopeadics..

[2] Gauthier G (1974). La Maladie de Freiberg ou 2ème maladie de Kohler: proposition d'un traitement de reconstruction au stade évolué de l'affection; In 48ème réunion annuelle de la SOFCOT. Rev Chir Orthop..

[3] Gauthier G, El Baz R (1979). Freiberg's infraction: a subchondral bone fatigue fracture: a new surgical traetement. Clin Orthop Relat Res..

[4] Smillie I (1927). Freiberg infraction. JBJS..

[5] Beyse N, Champagne H, Freneaux B (1991). Ostéonécroses aseptiques des têtes métatarsiennes et fractures de fatigues. Rhumatologie..

[6] Gauthier G (1996). La Maladie de Freiberg. In Cahiers Ens SOFCOT..

[7] Valtin B (1995). Les ostéotomies du col du 2ème métatarsien type Gauthier dans le syndrome de surcharge du 2ème rayon. Méd Chir Pied..

[8] Barouk LS (1996). Nouvelles ostéotomies de l'avant pied: description, insertion dans un concept thérapeutique global. In cahiers Ens SOFCOT..

[9] Deconinck JP (1974). La maladie de Freiberg ou ostéonécrose aseptique des têtes métatarsiennes, à propos de 47 cas opérés.

[10] Delagoutte JP (1981). Considération sur les métatarsalgies statiques. Rev Prat..

[11] Kinnard P, Lirette R (1991). Freiberg's disease and dorsiflexion osteotomy. JBJS..

